# Effect of Biannual Azithromycin to Children under 5 Years on the Carriage of Respiratory Pathogens among Children Aged 7–11 Years

**DOI:** 10.4269/ajtmh.22-0583

**Published:** 2022-12-19

**Authors:** Stephanie A. Brennhofer, Elizabeth T. Rogawski McQuade, Jixian Zhang, Suporn Pholwat, Suzanne Stroup, James A. Platts-Mills, Jie Liu, Eric R. Houpt

**Affiliations:** ^1^ Division of Infectious Diseases & International Health, University of Virginia, Charlottesville, Virginia;; ^2^ Department of Epidemiology, Rollins School of Public Health, Emory University, Atlanta, Georgia;; ^3^ School of Public Health, Qingdao University, Qingdao, China

## Abstract

In the MORDOR I trial, children under 5 years of age were randomized to receive biannual (every 6 months) azithromycin for 2 years in Niger, Malawi, and Tanzania. In 30 Nigerien communities, children aged 7–11 years, who were not enrolled in the MORDOR I trial to receive biannual azithromycin, were assessed for carriage of seven respiratory pathogens. We aimed to see whether there were effects on the carriage of these seven respiratory pathogens among 3,187 children aged 7–11 years living in the 30 communities via nasopharyngeal swabs collected at baseline (*N* = 1,066), as well as at year 1 (*N* = 1,019) and year 2 (*N* = 1,102)—each about 6 months after azithromycin or placebo treatment of children under age five. Most children were positive for *Haemophilus influenzae* (baseline: 83.8%; interquartile range [IQR]: 78.7–90.4) and *Streptococcus pneumoniae* (baseline: 82.9%; IQR: 74.2–86.8) at all time points regardless of treatment group. There were no differences in prevalence nor quantity of *H. influenzae* (prevalence ratio: 0.95; 95% CI: 0.90, 1.02), *S. pneumoniae* (prevalence ratio: 1.01; 95% CI: 0.96, 1.07), or any of the other respiratory pathogens in the treatment versus control groups at any time point. *S. pneumoniae* serotype 6AB (7.7%) and *Neisseria meningitidis* serotype W135 (24.9%) were the most prevalent serotypes detected among all positive *S. pneumoniae* and *N. meningitidis* samples, respectively. Biannual azithromycin did not reduce carriage of respiratory pathogens 6 months after the most recent round of biannual azithromycin among older nontreated children (aged 7–11 years) living in treatment communities.

## INTRODUCTION

In 2020, over half of the 5 million children under the age of 5 who died lived in sub-Saharan Africa.^1^ In high child mortality settings, innovative interventions are needed to prevent child deaths. The MORDOR (Macrolides Oraux pour Réduire les Décès avec un Oeil sur la Résistance) I trial, set in three sub-Saharan African countries, tested whether biannual azithromycin among children under 5 years of age could reduce child mortality. There was a 13.5% reduction in mortality in children under 5 years of age in communities who received biannual azithromycin compared with communities who did not receive azithromycin.^2^ This effect was most prominently seen in Niger (18.1% reduction) among all age groups and largely among children under 6 months of age in the two other sites.

An additional benefit to biannual azithromycin (every 6 months) in these high-risk communities could be an interruption of transmission of pathogens in the community, resulting in a reduction in infections among others who did not receive treatment. For example, biannual azithromycin for trachoma control programs in Ethiopia reduced ocular Chlamydia infections in the year following treatment in children^3^^,^^4^ and adults^4^ who lived in the treatment village but had not themselves received treatment. This is likely due to young children driving transmission in these settings, such that prevention of infections among young children reduced transmission in the community overall. It is unknown whether biannual azithromycin among younger children, who experience a disproportionate burden of respiratory illnesses, could prevent infections with respiratory pathogens in older children.

The purpose of this analysis was to determine whether there were indirect effects of the biannual azithromycin intervention on the carriage of respiratory pathogens among older children in the azithromycin treatment communities in the MORDOR I trial. These children themselves did not receive azithromycin nor placebo. However, if the under-5-year-olds were the dominant source of transmission for a particular pathogen, and if the effects of azithromycin were lasting, we would expect that carriage could be reduced in the older children.

## MATERIALS AND METHODS

This was an analysis of secondary outcomes in the Morbidity/Resistance substudy of the MORDOR I trial, which has been previously detailed.^2^ This substudy included 30 communities in Niger who were randomized to have < 5-year-old children receive azithromycin (*N* = 15) or a placebo (*N* = 15) every 6 months for 2 years from 2015 to 2017. At each phase of data collection (baseline, year 1, and year 2), 40 children aged 7–11 years were randomly selected from each community and had nasopharyngeal swabs collected. Samples were collected before children were treated with that round of biannual azithromycin; therefore, they were collected 6 months after the prior treatment. Ethical approval was obtained, and consent procedures were performed as detailed in the original manuscript.^2^ The original study can be found at ClinicalTrials.gov: NCT02048007.

Nasopharyngeal swabs were tested for respiratory pathogens and numerous serotypes of *Streptococcus pneumoniae* and *Neisseria meningitidis* by quantitative PCR (qPCR), with assays adapted from previous publications and validated on the TaqMan Array Card platform.^5^^–^^7^ Swabs were placed into DNA/RNA Shield (Zymo, Irvine, CA) spiked with external controls MS2 and PhHV. Nucleic acids were extracted with the QIAamp MinElute Virus Spin kit on QIAcube (Qiagen, Hilden, Germany) following the manufacturer’s instructions and eluted in 100 μL. One extraction blank was incorporated for up to 36 samples to rule out laboratory contamination. Forty-six microliters of the nucleic acid extract from each sample was mixed with 50 μL of AgPath One-Step RT-PCR buffer and 4 μL of enzyme mix, and then loaded onto each port of a TaqMan Array Card. The PCRs were performed on ViiA 7 or QuantStudio 7 real-time PCR systems, with the cycling conditions including the reverse transcription step at 45°C for 20 minutes, initial denaturation at 95°C for 10 minutes, 40 cycles of 95°C for 15 seconds, and 60°C for 1 minute. A quantification cycle (Cq) < 35 was considered positive.

The total number of positive swabs was calculated using the median and interquartile range (IQR) of the village prevalence. To quantify pathogen burden, we calculated the frequency of nasal swab positivity and average quantity of seven respiratory pathogens (*Haemophilus influenzae*, *Moraxella catarrhalis*, *Mycoplasma pneumoniae*, *N. meningitidis*, *Staphylococcus aureus*, *S. pneumoniae*, and *Streptococcus pyogenes*) at years 1 and 2. To estimate prevalence ratios for each pathogen comparing the treatment and control groups at years 1 and 2, we used Poisson regression with generalized estimating equations (GEEs) to account for clustering. We estimated pathogen quantity differences at years 1 and 2 via linear regression models with GEEs unadjusted and with adjustment for age in months, sex, and baseline cluster-level prevalence and quantity difference. Additionally, we calculated the percentage of positive samples per 1-year age groups for each of the seven respiratory pathogens. Sensitivity analyses were conducted by defining positives by higher-quantity infections using a qPCR Cq cutoff < 30. We quantified the prevalence of serotypes for higher-quantity *N. meningitidis* and *S. pneumoniae* detections (Cq < 30) by requiring the Cq of serotype genes to be within three cycles of the pathogen Cq.

## RESULTS

We included 3,187 distinct children and nasopharyngeal samples at three time points over a 2-year period: baseline (*N* = 1,066), year 1 (at 12 months of follow-up; *N* = 1,019), and year 2 (at 24 months of follow-up; *N* = 1,102). Children were sampled from 30 villages (*N* = 15 control; *N* = 15 treatment) with 1,610 and 1,577 children in the treatment and control villages, respectively. At year 1, samples were collected on average at 168 days (25th and 75th IQR: 157–200) for the treatment group and 201 days (25th and 75th IQR: 196–228) for the control group after the last treatment at 6 months of follow-up. At year 2, samples were collected on average at 130 days (25th and 75th IQR: 110–154) for the treatment group and 135 days (25th and 75th IQR: 124–168) for the control group after the last treatment at 18 months of follow-up. Among the 1,066 children sampled at baseline, 83.8% (IQR: 78.7–90.4) were positive for *H. influenzae*, followed by 82.9% (IQR: 76.6–87.1) for *S. pneumoniae* (Table 1). Prevalence of all other pathogens was less than 10% and the prevalences were comparable between treatment and control villages at baseline.

**Table 1:** Baseline characteristics among 1,066 asymptomatic children aged 7–11 years at baseline in the MORDOR I study

		Arm	
Characteristics	Overall	Treatment villages	Control villages
Total number of children included (*n*, %)	1,066	541 (50.8)	525 (49.2)
Male sex (*n*, %)	523 (49.1)	277 (51.2)	246 (46.9)
Age (years) (mean, SD)	8.8 (1.5)	8.9 (1.5)	8.8 (1.4)
Number of clusters (*n*, %)	30	15 (50.0)	15 (50.0)
Total number of positive swabs at baseline (village prevalence median, IQR)			
* H. influenzae*	83.8 (78.7–90.4)	82.9 (74.2–86.8)	86.8 (81.8–91.8)
* M. catarrhalis*	74.3 (60.5–77.2)	65.8 (58.5–76.6)	75.9 (69.1–77.9)
* M. pneumoniae*	0.0 (0.0–0.0)	0.0 (0.0–0.0)	0.0 (0.0–0.0)
* N. meningitidis*	6.0 (2.5–10.0)	7.5 (3.8–12.6)	3.3 (0.0–9.8)
* S. aureus*	7.5 (4.8–12.3)	10.0 (4.1–14.3)	6.9 (4.8–8.8)
* S. pneumoniae*	82.9 (76.6–87.1)	82.9 (76.8–85.8)	85.0 (77.4–88.2)
* S. pyogenes*	4.9 (2.6–7.8)	5.1 (2.6–13.5)	4.9 (2.7–7.5)

**Figure 1.**
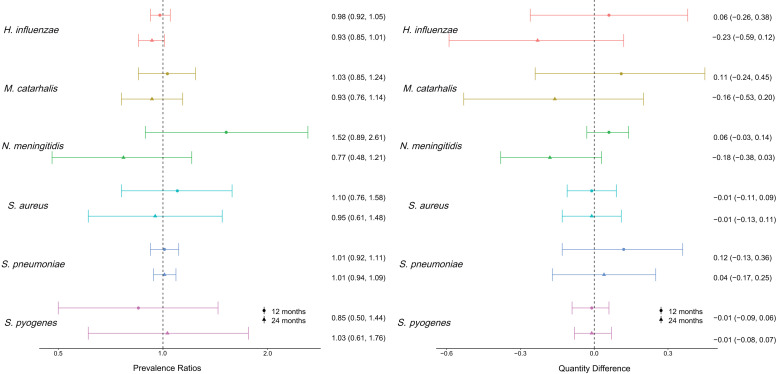
Comparing prevalence ratios and quantity differences of seven respiratory pathogens between the treatment and control groups among 2,121 children aged 7–11 years at years 1 and 2 in the MORDOR I study. Prevalence ratios compare prevalences at the later time points vs. baseline. Quantities are relative to baseline quantities.

At years 1 and 2 in the treatment and control groups, *H. influenzae* (year 1: 86.5% [IQR: 82.2, 94.3]; year 2: 87.7% [IQR: 78.1, 92.1]) and *S. pneumoniae* (year 1: 77.4 [IQR: 68.3, 80.4]; year 2: 83.1 [IQR: 79.2, 90.7]) were the most common pathogens Supplemental Table 1). These results were comparable to baseline. There was no difference in prevalence nor quantity at the sampling time points between the treatment and control groups for any of the pathogens overall or at any time point (Figure 1). On average, of the six pathogens tested for, −0.06 (95% CI: −0.21, 0.09) fewer pathogens were detected in the treatment group compared with the control group. Adjustment for age in months, sex, and baseline cluster-level prevalence and quantity difference did not alter the results (Supplemental Figure 1). Results were consistent across ages even when only higher-quantity infections were examined (Supplemental Figure 2). However, the incidence of *M. catarrhalis* decreased from ages 7 (64.4% positive swabs) to 11 years (48.7% positive swabs) and the incidence of *S. aureus* increased from ages 7 (10.9% positive swabs) to 11 years (17.8%).

**Figure 2.**
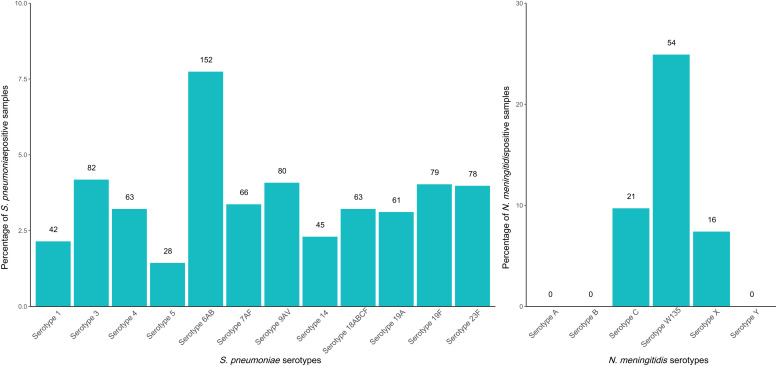
Prevalence of *S. pneumoniae* and *N. meningitidis* serotypes in positive samples among 3,187 children aged 7–11 years at baseline, year 1, and year 2 in the MORDOR I study. Numbers above columns represent raw numerators.

Three serotypes for *N. meningitidis* and 12 serotypes for *S. pneumoniae* were detected in samples from all three time points (Figure 2). *N. meningitidis*, serotype W135 was the most prevalent serotype detected, with 24.9% (*N* = 54/217 of *N. meningitidis* Cq < 30) positivity among all positive *N. meningitidis* samples followed by serotypes C (*N* = 21) and X (*N* = 16). *S. pneumoniae* serotype 6AB was the most prevalent serotype, with 7.7% (152/1,966) positivity among all positive *S. pneumoniae* samples.

## DISCUSSION

In this study, we found no evidence of an indirect effect of mass drug administration of azithromycin to younger children on respiratory pathogen carriage in older children, neither a reduction in pathogen prevalence nor pathogen quantity ∼6 months post the last treatment. In other words, this schedule of biannual azithromycin administration did not produce a measurable effect of nasopharyngeal carriage of bacterial respiratory pathogens among nontreated 7- to 11-year-old children.

The ability of a drug to offer indirect protection is likely due to multiple factors such as the potency of the drug administered, pathogen and transmission pathways, baseline prevalence in the community, administration frequency, baseline resistance, average duration, frequency of infection, and sample collection time after drug administration. One potential explanation for this null finding is that younger children are not critical reservoirs for transmission of these respiratory pathogens to older children. Another possibility is that the intensity or duration of biannual azithromycin was not high enough to alter transmission in the community. Another possibility is that the time lag was too long between drug administration and nasopharyngeal testing to see an effect. For instance, it is known from children with invasive disease with *S. pneumoniae* and/or *H. influenzae* that treatment with antibiotics clearly reduces nasopharyngeal colonization; however, the effect is temporary.^8^ In one study, carriage returned to baseline in about half of cases by 3 weeks, and in another was only reduced by 27% at 2 months.^9^^–^^11^

The carriage prevalences were generally expected. In the Malawi site of MORDOR, a subset of the younger children were tested for *S. pneumoniae* by nasopharyngeal culture at similar time points after biannual azithromycin and ∼10–20% were positive (reduced from a pretreatment baseline of ∼40–50%).^12^^,^^13^ The 80% prevalence of pneumococcal carriage we detected in older children is higher, but reasonably expected because we used qPCR, which is generally more sensitive than culture.^14^ The prevalence was also higher than that observed in other studies in the region, which ranged from 20% to 65%.^15^^–^^18^ Whereas vaccine studies in young children show that they are important reservoirs of pneumococcus transmission to older individuals in high-income settings such as the United States,^19^ in sub-Saharan Africa there is less indirect protection against pneumococcal disease or carriage.^15^

The high rates of colonization of *M. catarrhalis* and *H. influenzae*, and variable rates with *S. aureus*, are consistent with other studies from Burkina Faso and Kenya.^18^^,^^20^ We also noticed a trend toward a decrease in colonization with age, which is well described.^18^^,^^20^

As for the molecular typing results, Niger currently vaccinates against *H. influenzae* (Hib3) and *S. pneumoniae* (pneumococcal conjugate vaccine; PCV-13) as part of their national immunization schedule. We do not have village-level data on vaccine coverage but, while this study was conducted, national coverage of the three-dose PCV-13 vaccine rose from 13% to 76% in 2016, while coverage for Hib3 vaccine remained steady at around 80%.^21^ PCV-13 contains the pneumococcal serotype 6AB and reductions have been reported with vaccination. In this study, 6AB was found to be the most common serotype (of the serotype assays that could be accommodated on the card). Substantial rates of 6A and 6B, even after vaccination, have been noted previously.^22^

Niger has also introduced the group A meningococcal conjugate vaccine (PsA-TT, MenAfriVac).^23^ In our study, the most prevalent serotype for *N. meningitidis* was W135 (*N* = 54) with no instances of serotype A. This is consistent with other studies, which have found very little^24^ to no evidence^25^ of serotype A and higher rates of serotypes W and C.^24^^–^^26^

In summary, there was no apparent reduction in *S. pneumoniae* and *H. influenzae* nasopharyngeal carriage in older children via treatment in younger children. A high burden of *S. pneumoniae* and *H. influenzae* nasopharyngeal carriage remained. We observed no instances of *N. meningitidis* serotype A. Use of this TaqMan Array Card provided an efficient and high-throughput tool to screen and surveil for multiple respiratory pathogens of public health importance.

## Supplemental files


Supplemental materials
