# Supplementary material for: Effect of Biannual Azithromycin to Children under 5 Years on the Carriage of Respiratory Pathogens among Children Aged 7–11 Years
Source: Am J Trop Med Hyg. 2022 Dec 19;108(2):428–32. doi: 10.4269/ajtmh.22-0583 (PMC9896336; doi:10.4269/ajtmh.22-0583)

Supplementary materials

Effect of mass drug administration with azithromycin to children under 5 years on the carriage of respiratory pathogens among children aged 7-11 years

Stephanie A Brennhofer, Elizabeth T Rogawski McQuade, Jixian Zhang, Suporn Pholwat,

Suzanne Stroup, James A Platts-Mills, Jie Liu, Eric R Houpt

Correspondence to: Elizabeth T Rogawski McQuade
Email: erogaws@emory.edu

Supplemental Table 1. Frequency and quantity of nasal swab positivity for seven respiratory pathogens among 2,121 children ages 7-11 years at phases 12 and 24 months in the MORDOR study.

Pathogen	Swabs positive at 12 months (village prevalence median, IQR)		Swabs positive at 24 months (village prevalence median, IQR)		Prevalence ratios at 12 and 24 months (95% CI)	Average quantity of pathogen detected at 12 months (mean, SD)[d]		Average quantity of pathogen detected at 24 months (mean, SD)[d]		Quantity difference at 12 and 24 months (95% CI)
	Treatment	Control	Treatment	Control		Treatment	Control	Treatment	Control	
H. influenzae[a]	83.9 (82.1, 94.3)	89.7 (83.5, 93.4)	83.8 (75.7, 88.5)	90.0 (83.6, 94.7)	0.95 (0.90, 1.02)	2.63 (1.65)	2.54 (1.58)	2.46 (1.69)	2.68 (1.59)	-0.09 (-0.36, 0.19)
M. catarrhalis[b]	46.2 (41.2, 58.9)	48.8 (45.3, 53.6)	53.7 (40.9, 62.3)	59.0 (46.8, 63.7)	0.97 (0.84, 1.13)	1.19 (1.73)	1.04 (1.61)	1.22 (1.69)	1.40 (1.79)	-0.03 (-0.29, 0.24)
M. pneumoniae[c]	0.0 (0.0, 0.0)	0.0 (0.0, 0.0)	0.0 (0.0, 0.0)	0.0 (0.0, 0.0)	--	-0.30 (0.00)	-0.30 (0.07)	-0.30 (0.08)	-0.30 (0.00)	--
N. meningitidis	6.9 (5.0, 12.7)	5.3 (2.6, 9.8)	9.3 (7.4, 16.9)	12.9 (10.2, 21.7)	0.89 (0.59, 1.34)	-0.13 (0.65)	-0.18 (0.55)	0.01 (0.95)	0.19 (1.20)	-0.07 (-0.18, 0.05)
S. aureus	13.9 (9.0, 24.3)	13.8 (10.4, 21.2)	14.3 (7.6, 18.5)	17.5 (13, 19.8)	1.03 (0.72, 1.47)	-0.09 (0.54)	-0.07 (0.61)	-0.08 (0.58)	-0.06 (0.59)	0.00 (-0.10, 0.10)
S. pneumoniae	77.8 (69.3, 80.2)	76.2 (68.8, 80.5)	83.3 (78.8, 92.5)	82.9 (79.7, 86.7)	1.01 (0.96, 1.07)	1.54 (1.31)	1.40 (1.26)	1.80 (1.24)	1.76 (1.25)	0.08 (-0.07, 0.24)
S. pyogenes	7.1 (3.1, 10.3)	7.1 (2.5, 11.6)	9.8 (3.1, 12.0)	7.5 (4.2, 11.2)	0.94 (0.63, 1.41)	-0.19 (0.46)	-0.17 (0.47)	-0.18 (0.43)	-0.17 (0.47)	-0.01 (-0.06, 0.04)

H. influenzae = *Haemophilus influenzae*. *M. catarrhalis* = *Moraxella catarrhalis*. *M. pneumoniae* = *Mycoplasma pneumoniae*. *N. meningitidis* = *Neisseria meningitidis*. *S. aureus* = *Staphylococcus aureus*. *S. pneumoniae* = *Streptococcus pneumoniae*. *S. pyogenes* = *Streptococcus pyogenes*. [a] N=2027. [b] N=2092. [c] N=2120. [d] N=2125. [d] Ct values transformed to the log10 scale to indicate log10 copies. After transformation, all 0 values were set to half of the limit of detection at log10(0.5).

Supplemental Figure 1. Adjusted model comparing prevalence ratios and quantity differences of seven respiratory pathogens between the treatment and control groups among 2,121 children ages 7-11 years at phases 12 and 24 months in the MORDOR I study (N=2121). Model adjusted for: age in months, sex, and phase 0 cluster-level prevalence and quantity difference. *H. influenzae* = *Haemophilus influenzae*. *M. catarrhalis* = *Moraxella catarrhalis*. *M. pneumoniae* = *Mycoplasma pneumoniae*. *N. meningitidis* = *Neisseria meningitidis*. *S. aureus* = *Staphylococcus aureus*. *S. pneumoniae* = *Streptococcus pneumoniae*. *S. pyogenes* = *Streptococcus pyogenes*.

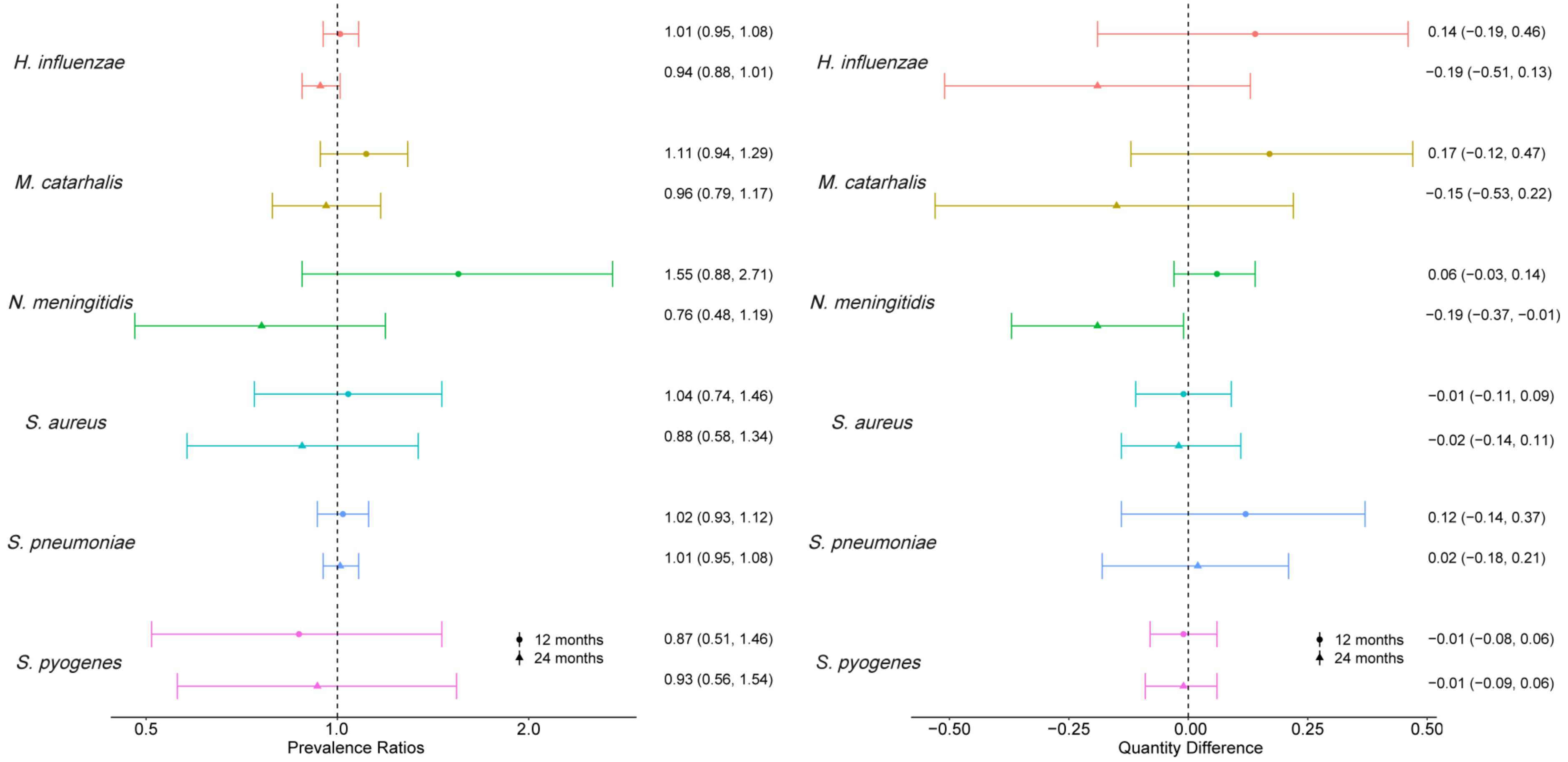

Supplemental Figure 2. Percentage of positive respiratory pathogen samples per year of age with a Ct cut off <35 and <30 among 3,187 children ages 7-11 years at phases 0, 12, and 24 months in the MORDOR I study. *H. influenzae* = *Haemophilus influenzae*. *M. catarrhalis* = *Moraxella catarrhalis*. *M. pneumoniae* = *Mycoplasma pneumoniae*. *N. meningitidis* = *Neisseria meningitidis*. *S. aureus* = *Staphylococcus aureus*. *S. pneumoniae* = *Streptococcus pneumoniae*. *S. pyogenes* = *Streptococcus pyogenes*.

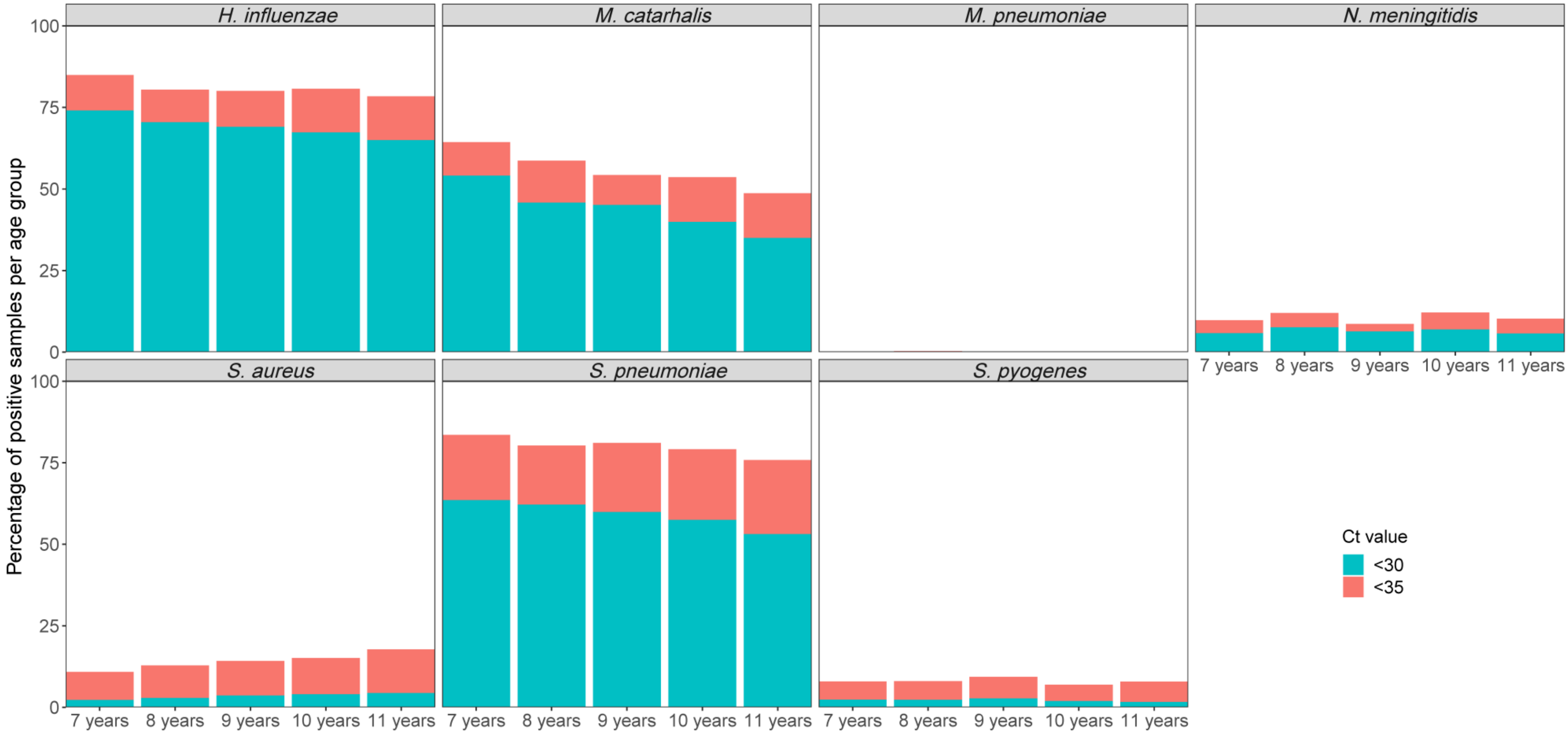

Supplement: Supplementary file 1 [file tpmd220583.SD1.pdf]
